# Molecular characteristics, clinical significance, and cancer immune interactions of cuproptosis and ferroptosis-associated genes in colorectal cancer

**DOI:** 10.3389/fonc.2022.975859

**Published:** 2022-09-05

**Authors:** Yang Li, Ru-yao Wang, Yu-jiao Deng, Shao-hua Wu, Xinti Sun, Hong Mu

**Affiliations:** ^1^ Department of Clinical Laboratory, Tianjin First Central Hospital, School of Medicine, Nankai University, Tianjin, China; ^2^ Blood Transfusion Department, Qingdao Women and Children’s Hospital, Qingdao, China; ^3^ Department of Clinical Training and Teaching , Tianjin University of Traditional Chinese Medicine, Tianjin, China; ^4^ Department of Thoracic Surgery, Tianjin Medical University General Hospital, Tianjin, China

**Keywords:** cuproptosis, ferroptosis, colorectal cancer, nomogram, overall survival

## Abstract

**Objective:**

To systematically analyze the expression of cuproptosis and ferroptosis genes and their impact on the development, prognosis, tumor microenvironment (TME), and treatment response in colorectal cancer (CRC) patients

**Methods:**

We systematically evaluated 33 cuproptosis and ferroptosis-related genes and comprehensively identified the correlations between cuproptosis and ferroptosis-related genes and transcriptional patterns, prognosis, and clinical features. Three distinct subgroups were identified in CRC using the TCGA database and the GEO database. We next assessed the relationship between the molecular features, prognostic significance, and clinical indicators of the prognostic genes in the cuproptosis and ferroptosis-related gene clusters. In addition, a PAC_score, which accurately predicted the prognosis of CRC patients and the efficacy of immunomodulatory mAbs, was obtained.

**Results:**

Patients in the low expression group (low expression of cuproptosis and ferroptosis-related genes) had a longer survival compared to the high expression group. We identified two distinct prognosis-associated molecular subtypes and observed an association between clinical information and prognosis. The enrichment analysis of differential genes associated with prognosis showed that the main enrichment was related to biological processes such as metastasis and metabolism. Next, the PCA_score for predicting overall survival (OS) was established and its reliable predictive value in CRC patients was confirmed. Furthermore, highly reliable nomogram was created to facilitate the clinical feasibility of the PCA_score. It was found that the immunomodulatory mAbs, PD-L1 and CTLA4 were highly expressed in the low PCA_score score group with statistically significance.

**Conclusion:**

Overall, the PCA scores of prognostic differential genes in the cuproptosis and ferroptosis-related gene clusters were strongly associated with clinical characteristics, prognosis, and immunotherapy in CRC patients. This data may promote further exploration of more effective immunotherapy strategies for CRC.

## Introduction

Colorectal cancer (CRC) is the most common malignancy worldwide, followed by lung and breast cancer. It is also the fourth common malignancy and the second leading cause of cancer death in the United States (1). Surgery is still considered the best treatment approach for primary tumor; yet, at the time of diagnosis, most patients present with advanced-stage tumor, losing their chance to undergo surgery. Considering the high morbidity and mortality of CRC, there is an urgent need to develop more efficient prognostic models. It is necessary to develop valuable biomarkers that can classify patients with different characteristics into distinct groups and predict the effect of immunotherapy. Chemoteprha, radiotherapy, and immunotherapy are the most popular methods to treat advanced-stage CRC. Approximately 66% and 61% of stage II and III colon and rectal patients, respectively, received adjuvant chemotherapy and/or radiation therapy (2). Furthermore, 54% of patients relapse after neoadjuvant treatment (3). Immunotherapy is an emerging treatment for many types of tumors, and many studies have confirmed its effectiveness on tumors (4). Common immunotherapy strategies include ICP inhibitors (ICIs), therapeutic antibodies, and cell therapy (5, 6). Anti-PD-1 therapy has shown a good response in some human malignancies, including melanoma, non-small cell lung cancer, and renal cell carcinoma.

Previous studies have shown that the tumor microenvironment (TME) is responsible for aggressive tumor behavior and affects tumor response to immunotherapy (7, 8). The TME is composed of multiple factors, including tumor cells, blood vessels, infiltrating immune cells, stromal cells, tissue fluid, and cytokines.

Copper is an indispensable trace element involved in various biological processes. Cuproptosis is a new form of programmed cell death during which copper bind directly to fatty acylated components of the tricarboxylic acid (TCA) cycle, leading to toxic protein stress and ultimately cell death (9). Ferroptosis is a regulated form of cell death primarily dependent on iron-mediated oxidative damage and subsequent cell membrane damage (10). Ferroptosis has dual tumor-promoting and tumor-suppressive roles in tumorigenesis, which depend on the release of damage-associated molecular patterns and the activation of ferroptosis-induced immune responses within the tumor microenvironment (TME) (11). Yet, the relationship between cuproptosis combined with ferroptosis-related genes and the tumor microenvironment is not fully understood.

In this study, we systematically analyzed the expression of cuproptosis and ferroptosis genes and their impact on the development, prognosis, TME, and treatment response of CRC patients. We hope that this study will contribute to the development of viable CRC immunotherapies.

## Materials and methods

### Data collection

Data on cuproptosis and ferroptosis-related genes, RNA expression data, somatic mutation data, CNV files, and clinical information of CRC patients were obtained from The Cancer Genome Atlas (TCGA) database (https://portal.gdc.cancer.gov//, accessed on 15 March 2022). GSE39582 from the GEO repository was utilized to acquire clinical parameters and normalized gene expression data. These data were generated using the platform of Affymetrix Human Genome U133 Plus 2.0 Array (GPL570). Some samples were excluded from further analysis due to the lack of important clinicopathological or survival information. A total of 278 cuproptosis and ferroptosis-related genes were obtained from the world’s first database of ferroptosis regulators and markers and ferroptosis-disease associations (http://www.zhounan.org/ferrdb/legacy/operations/download.html) and the Tsvetkov P Team (9).

### Consensus cluster analysis of cuproptosis and ferroptosis-related genes

Through the k-means algorithm, consensus clustering was employed to define different angiogenesis-related patterns. The quantity, as well as consistency of clusters, were built by the consensus clustering algorithm, which is available in the “ConsensuClusterPlus” package (12). To identify differences in biological functions of copper death and ferroptosis-related genes, gene set variation analysis (GSVA) was performed using the KEGG gene set (c2.cp.kegg.v7.2.gmt) (13).

### Association of molecular patterns with clinical features and prognosis of CRC

To determine the clinical significance of clusters generated by consensus clustering, we investigated associations between molecular patterns and clinical features, as well as survival outcomes. Clinical variables included age, gender, T-stage and N-stage. In addition, Kaplan-Meier analyses obtained by the “survival” and “survminer” software packages were used to assess differences in OS between the different modalities (14).

### Identification and functional enrichment analysis of DEGs

To identify DEGs in different subgroups of cuproptosis and ferroptosis-related genes, we used the ‘limma’ package with |log2-fold change (FC)| ≥ 1 and p-value < 0.001. These DEGs were used for GO and KEGG analysis using the “clusterProfiler” package (15).

### Construction of prognostic gene scores associated with cuproptosis and ferroptosis

Expression data of DEGs from different cuproptosis and ferroptosis-related genes clusters were normalized in CRC samples and intersecting genes were selected. The differential assessment indicated that there were 786 DEGs between the three cuproptosis and ferroptosis-related gene clusters. We then performed a univariate Cox regression (uniCox) analysis of DEGs. Survival-related genes were retained for further analysis. We also performed principal component analysis (PCA) to generate prognostic related gene scores using the following algorithm: PCA_score= expression of a gene [1] ×corresponding coefficient [1] + expression of a gene [2] ×corresponding coefficient [2] + expression of gene [n]× corresponding coefficient [n].

### Clinical correlation analysis of the DEGs PCA _score

The correlation of PCA_score with clinical variables was investigated. We also investigated the expression of immunomodulatory monoclonal antibodies, PD-L1 and CTLA4 in high and low PCA_score groups.

### Creation of predictive nomogram

The nomogram has been described as a valuable clinical predictor of risk scores and other clinicopathological features in CRC patients, particularly with regard to 1-, 3-, and 5-year OS. We further performed calibration curve analysis to confirm the clinical reliability of the established nomogram.

### Statistical analysis

All statistical analyses and plots were processed by SPSS 25.0 and R software (version 4.1.0). The PCA_group, age, gender, and tumor stage were used to set up a nomogram for the 1-, 3-, and 5-year OS and calibration curves based on the Hosmer–Lemeshow test to illustrate whether the predictive outcome showed good consistency with the practical outcome by the rms R package. All statistical tests were two‐sided and P values of <0.05 were considered statistically significant.

## Results

### Genetic mutation landscape of cuproptosis and ferroptosis-related genes in CRC

**Figure 1:**
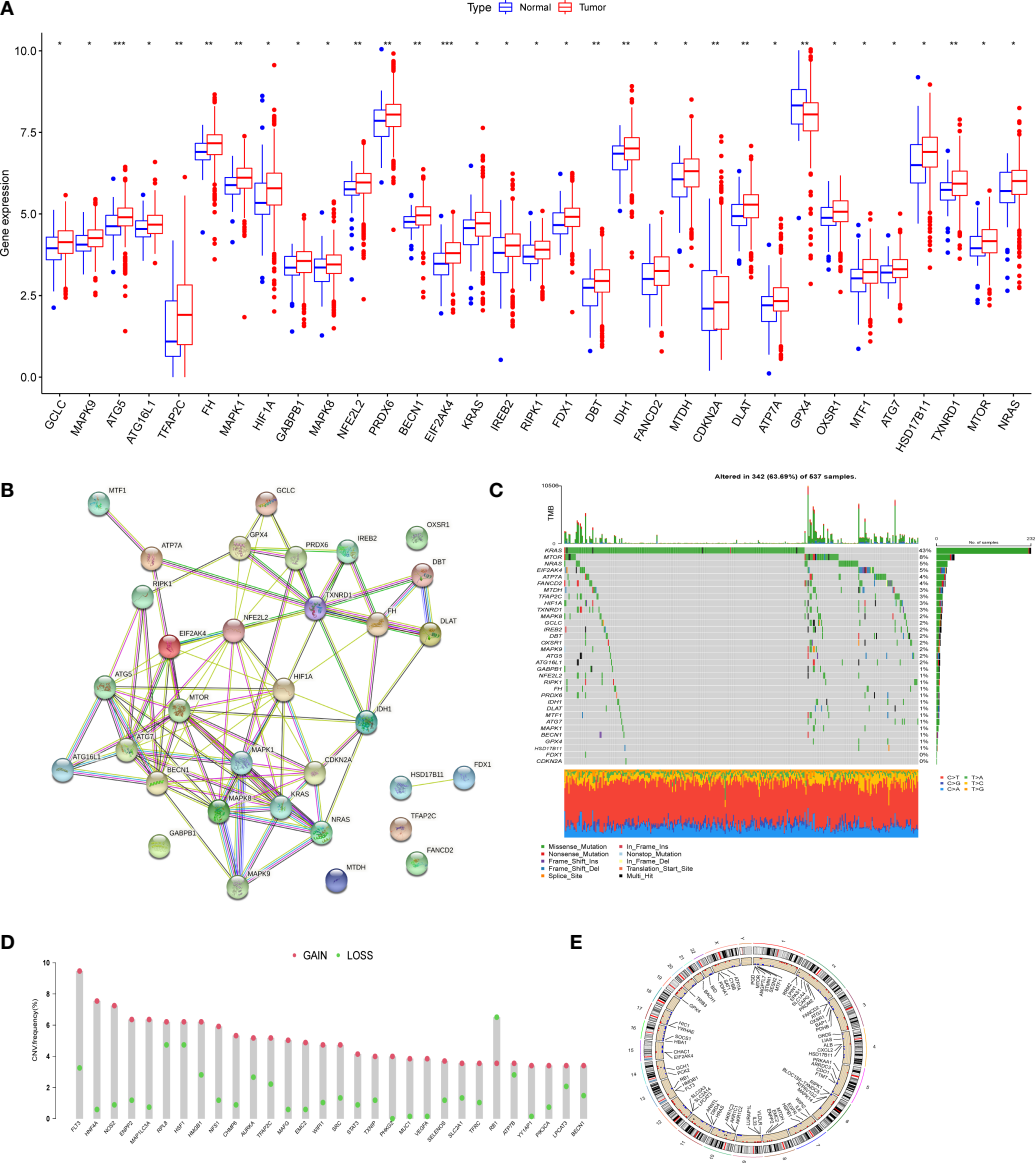
Genetic mutational landscape of cuproptosis and ferroptosis-related genes in CRC. **(A)** Expression dristribution of DEGs between tumor tissue and normal tissue. **(B)** The PPI network acquired from the STRING database among the DEGs. **(C)** Genetic alteration on a query of cuproptosis and ferroptosis-related genes. **(D)** Frequencies of CNV gain, loss, and non-CNV among cuproptosis and ferroptosis-related genes. **(E)** Circus plots of chromosome distributions of cuproptosis and ferroptosis-related genes. *P < 0.05; **P < 0.01; ***P < 0.001.

We first determined the expression levels of 278 cuproptosis and ferroptosis-related genes in tumor specimens and normal specimens using the TCGA dataset and found 33 DEGs, most of which were abundant in tumor samples (**Figure 1A**). A protein-protein interaction (PPI) analysis was established through the string website, revealing the interaction of DEGs; ATP7A、DLAT、CDKN2A、ATG5 and ATG7 were hub genes (**Figure 1B**). Then, we determined the incidence of CNVs and somatic mutations in genes associated with differential expression of cuproptosis and ferroptosis in CRC. As shown in **Figure 1C**, 342 of the 537 CRC samples (63.69%) had gene mutations, and the results showed that KRAS was the gene with the highest mutation rate, followed by MTOR and NRAS. In addition, we explored CNV mutation rates, which showed that 33 cuproptosis and ferroptosis-related genes exhibited marked CNV alterations (**Figure 1D, E**). We concluded that CNVs might have a regulatory role in the expression of cuproptosis and ferroptosis-related genes.

### Generation of cuproptosis and ferroptosis-related genes subsets in CRC

**Figure 2:**
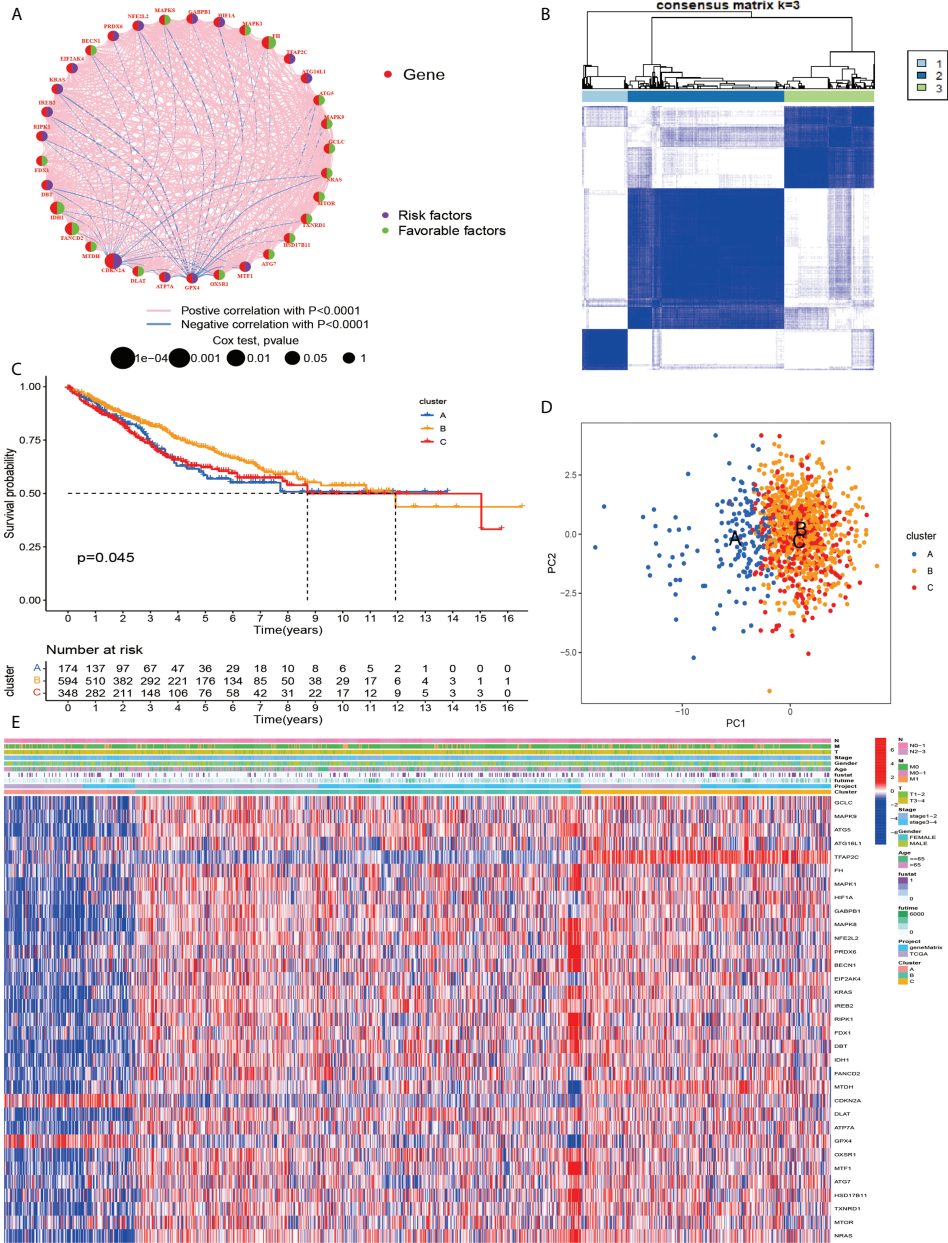
**(A)** Related networks, including AAG cuproptosis and ferroptosis-related in the TCGA cohort. **(B)** A consensus matrix heatmap three clusters (k = 3) and their associated regions. **(C)** Relationship between PCA analysis and subgroup transcriptome data. **(D)** Univariate analysis showed that 33 cuproptosis and ferroptosis-related genes were associated with OS. **(E)** Differences in clinocopathological characteristics and cuproptosis and ferroptosis-related genesexpression levels among three different subgroup.

To investigate the relationship between cuproptosis and ferroptosis-related genes and tumorigenesis, a total of 1082 CRC patients from TCGA and GSE39582 were included in this study. The correlation network of cuproptosis and ferroptosis-related genes and their regulatory relationships in CRC patients are shown in **Figure 2A**. To further determine the relationship between the expression patterns of cuproptosis and ferroptosis-related genes and CRC subtypes, we performed a consensus clustering analysis to classify CRC patients according to the expression levels of these cuproptosis and ferroptosis-related genes. The optimal clustering variable was 3 (**Figure 2B**) and CRC patients in the entire cohort were well dispersed in cluster A (n = 175), cluster B (n = 602) and cluster C (n =349). Furthermore, OS times for the three clusters were discussed and significant survival differences were observed (**Figure 2C, D**). We found that cuproptosis and ferroptosis-related genes were under-expressed in cluster A, but CDKN2A and GPX4 showed opposite results (**Figure 2E**).

### Characteristics of the TME in different subgroups

**Figure 3:**
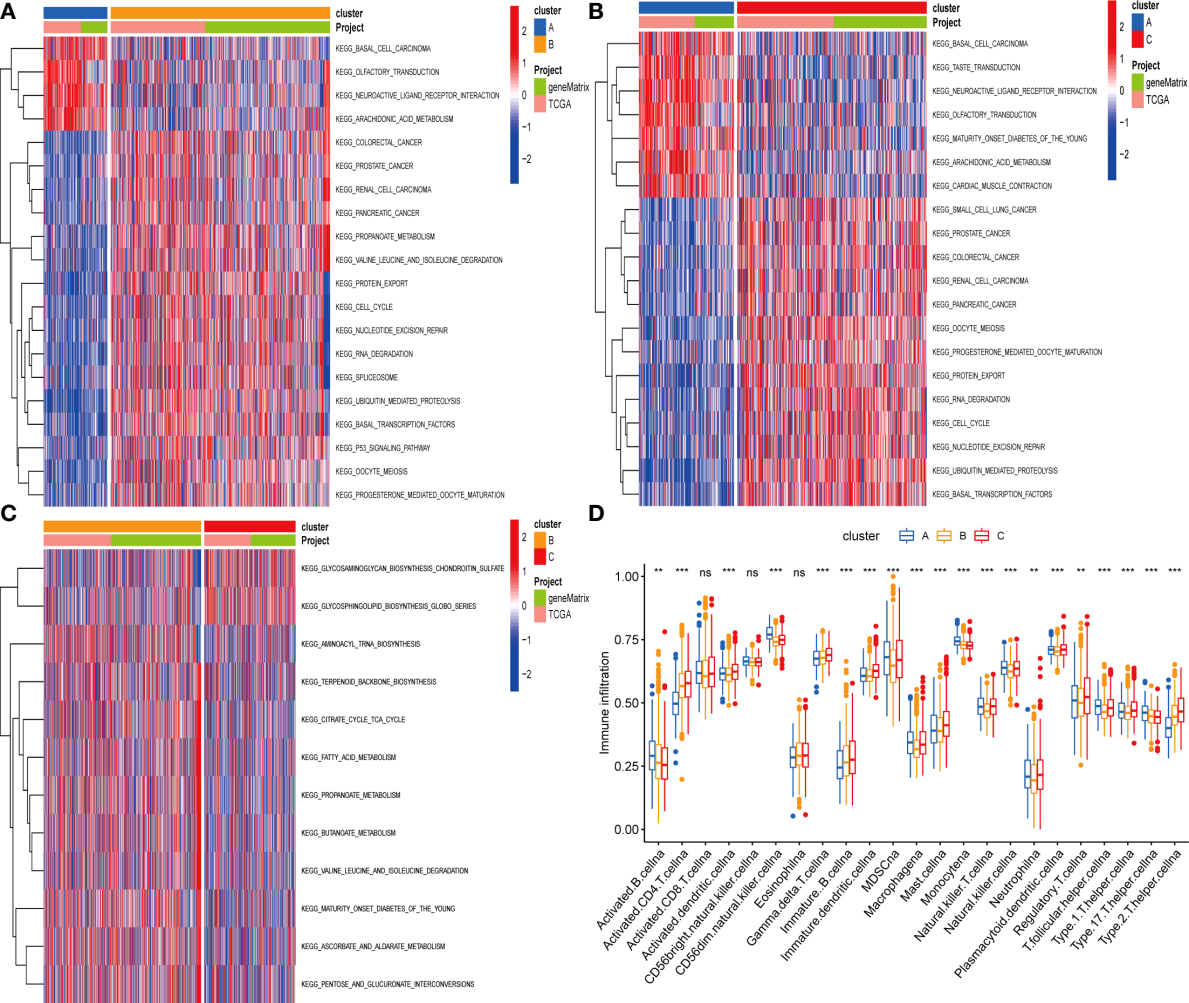
**(A–C)** GSVA of biological pathways between three distinct subgroups. **(D)** Abundance of 23 infiltrating immune cell types in the two GC subgroups. **P < 0.01; ***P < 0.001. ns, not significant.

According to the results of GSVA analysis, cluster A is involved in cancer-related pathways (colorectal cancer, prostate cancer, renal cell cancer and other cancers) and fatty acid and protein transport systems (**Figures 3A–C**). To determine the relationship between cuproptosis and ferroptosis-related genes and TME in CRC, we explored the infiltration levels of 23 human immune cell subsets in three clusters using the CIBERSORT algorithm. As shown in **Figure 3D**, significant differences in the enrichment of most immune cells were observed between the three clusters. CD56dim.natural.killer.cell, Immature.dendritic.cell, MDSC, Mast.cell, Regulatory.T.cell, etc. were lowly expressed in cluster B. Survival analysis among the three groups shows that cluster B survives longer.

### Identification of gene subgroups based on DEGs

**Figure 4:**
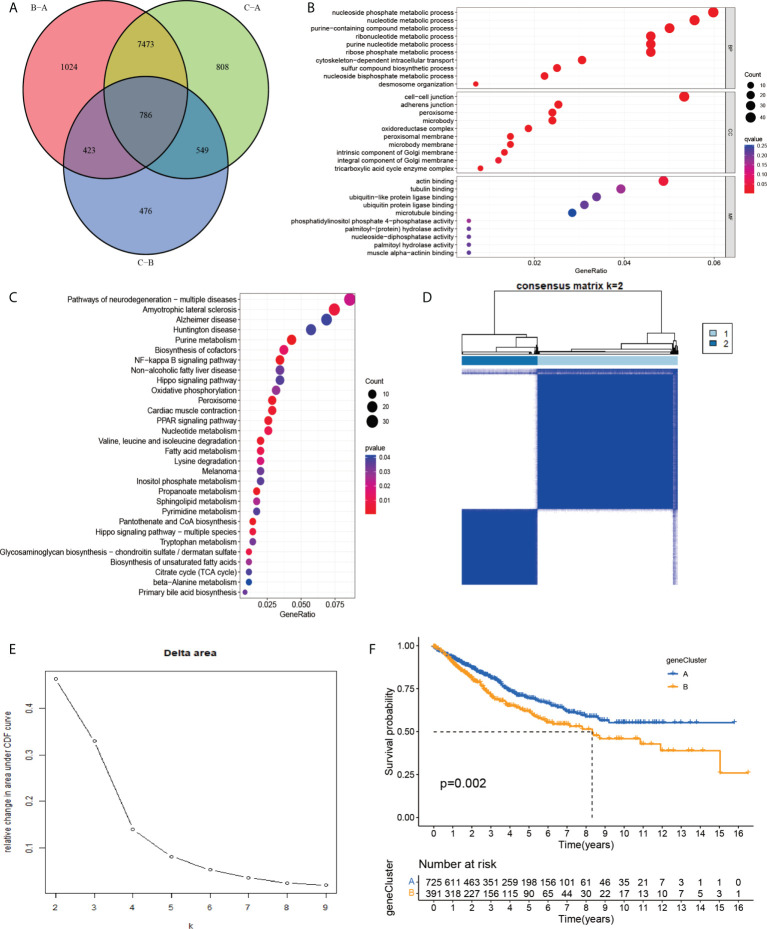
**(A)** A cluster of genes to cuproptosis and ferroptosis, a Venn diagram of differential genes. **(B, C)** GO and KEGG enrichment analysis of DEGs in three subgroups of cuproptosis and ferroptosis-related genes. **(D, E)** Consensus matrix heatmap defining two clusters (k = 2) and their correlation area. **(F)** Univariate analysis showed that 231 prognotic genes were associated with OS.

To investigate the potential biological activities of the cuproptosis and ferroptosis-related gene subsets, we obtained 786 DEGs related to the cuproptosis and ferroptosis-related gene subsets using the “limma” and performed functional enrichment analysis (**Figure 4A**). DEGs associated with these cuproptosis and ferroptosis-related gene subsets were mainly enriched in biological processes related to metabolism and metastasis (**Figures 4B, C**). Univariate survival analysis found that 231 differential genes were associated with prognosis (P<0.05). The results of the analysis showed that the optimal clustering variable was 2 for 231 differential genes associated with prognosis (**Figures 4D, E** **)**, and CRC patients in the whole cohort were well dispersed in cluster A (n = 732), cluster B (n=394). Kaplan-Meier analysis demonstrated that patients in cluster B had the shortest OS time (**Figure 4F**).

### Development and validation of the prognostic cuproptosis and ferroptosis-related genes score

**Figure 5:**
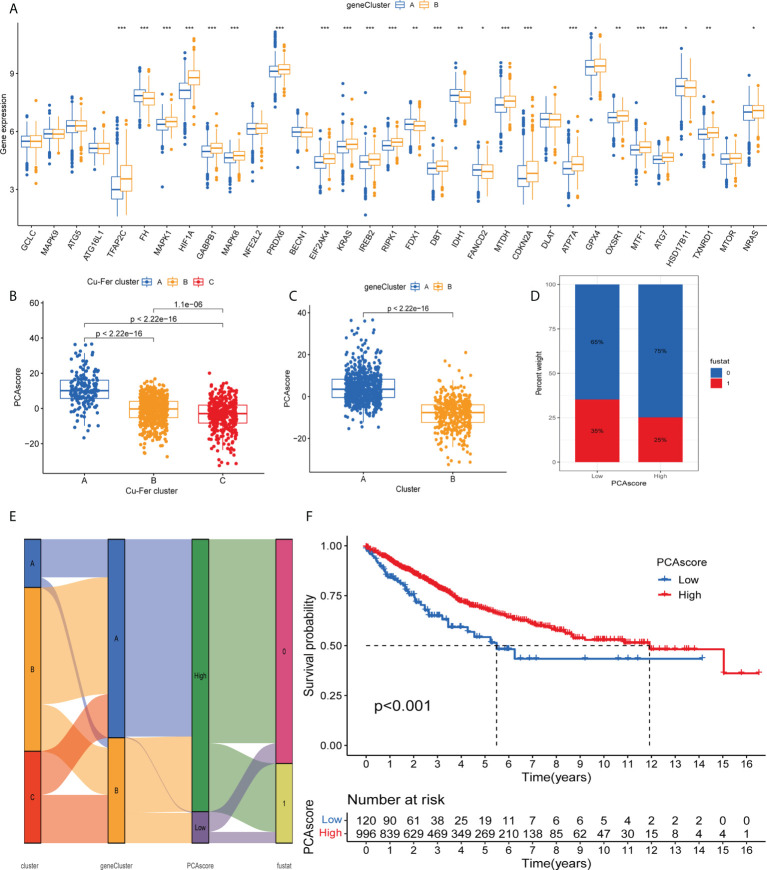
**(A)** Differences in the expression of 33 cuproptosis and ferroptosis-related genes among the two gene cluster. **(B)** Differences in cuproptosisand ferroptosis-related genes_score between thr three clusters. **(C)** Differences in cuproptosis and ferroptosis-related genes_score betweenthe two gene clusters. **(D)** Grouped by PCA score of cuproptosis and ferroptosis-related genes, patient survival status. **(E)** Alluvil diagram ogsubgroup distributions in groups with different cuproptosis and ferroptosis-related genes_scores and clinical outcomes. **(F)** Kaplan-Meieranalysis of the OS between the two groups. *P < 0.05; **P < 0.01; ***P < 0.001.

As expected for the cuproptosis and ferroptosis-related genes subgroups, the cuproptosis and ferroptosis-related gene clusters showed significant differences (**Figure 5A**). We discovered a substantial difference in the cuproptosis and ferroptosis-related genes _score of the cuproptosis and ferroptosis-related genes clusters and gene clusters (**Figures 5B, C** **)**. The **Figure 5D** shows the subgroups of PCA scores, and the distribution status of patient survival and death. **Figure 5A** displays the patients’ distribution in the three cuproptosis and ferroptosis-related genes clusters, two gene clusters, and two cuproptosis and ferroptosis-related genes_score groups (**Figure 5E**). We observed that the DEGs_score in gene cluster A was significantly higher than in gene cluster B. Based on the abovementioned survival analysis, we identified that higher PCA scores were correlated with better survival (**Figure 5F**).

### Clinical correlation analysis of the DEGs PCA score

**Figure 6:**
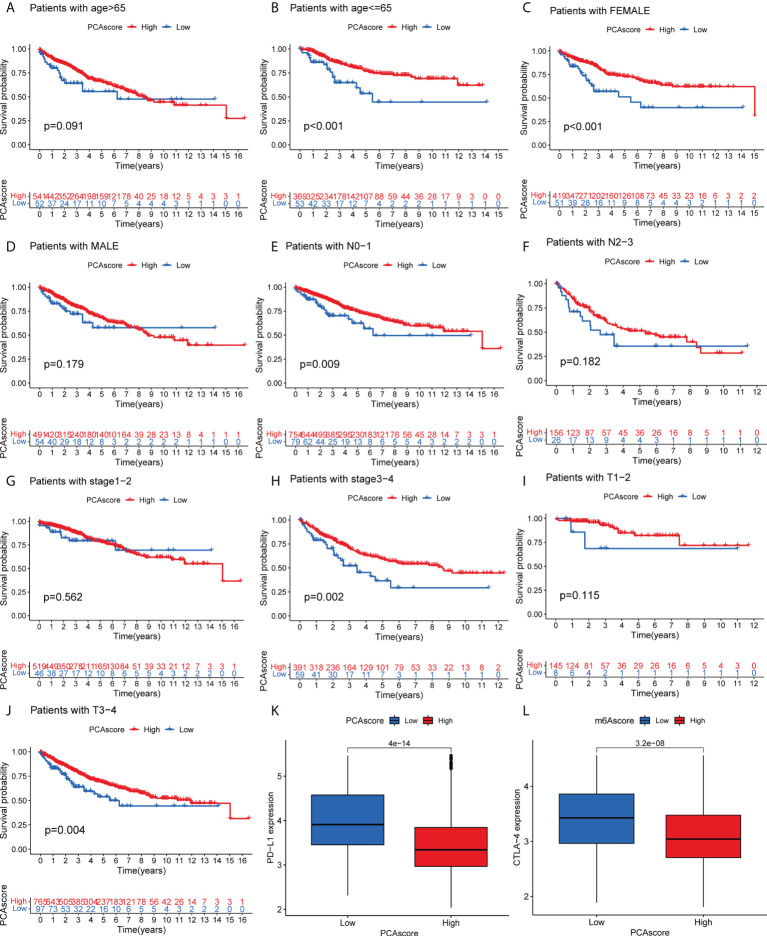
**(A–J)** Survival of PCA score subgroups and clinical information. **(K)** Expression of PD-L1 in PCA score subgroups. **(L)** Expression of CTLA-4 in PCA score subgroups. .

As shown in **Figure 6**, survival status was related to age <= 65, female, N0-1 stage, Stage III-IV, and TIII-IV stage in the PCA grouping. PD-L1 expression was significantly high in the low PCA score group. Similarly, CTLA-4 was highly expressed in the low PCA score group, and the difference was statistically significant. High expression of both PD-L1 and CTLA-4 can inhibit the activity of T cells, which may lead to a lack of immune response in cancer patients, resulting in immune evasion of cancer cells and promoting cancer cell proliferation. The survival time of the PCA low-score group was shorter than that of the high-level group.

### Multidimensional validation

**Figure 7:**
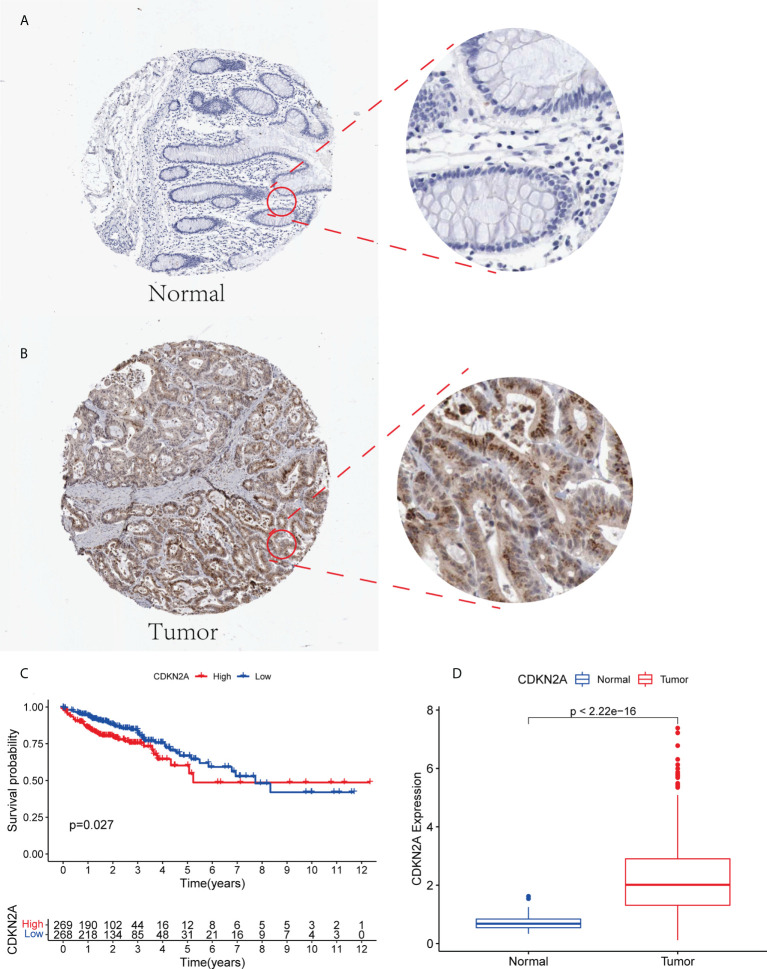
**(A, B)** The expression level of CDKN2A in normal colorectal tissue and CRC in HPA database. **(C)** Kaplan-Meier survival analysis based on the expression levels of CDKN2A in CRC in TCGA database. **(D)** The expression of CDKN2A in normal group and tumor group.

CDKN2A was highly expressed in tumor patients and correlated with prognosis. We performed a multidimensional validation in the HPA database (**Figure 7D**). The HPA database was exploited to evaluate the protein expression level of CDKN2A. We observed that CDKN2A was positively stained in the nuclear of colorectal tumor cells compared with the normal tissue (**Figures 7A, B**). The high expression level of CDKN2A was significantly associated with poor OS in CRC patients (**Figure 7C**).

### Construction of a nomogram to predict patients’ prognosis

**Figure 8:**
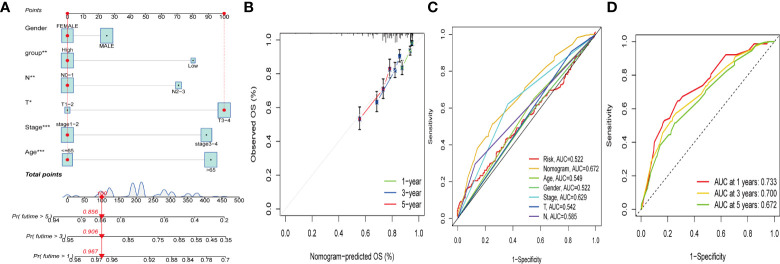
Construction and validation of a nomogram. **(A)** Nomogram for predicting the 1-, 3-, and 5-years OS of CRC patients in the entire cohort. **(B)** ROC curves for predicting the 1-, 3-, and 5-year ROC curves in the entire cohort. **(C, D)** The time-dependent ROC curves of the nomograms compared for 1-, 3-, and 5-years OS in GC, respectively.

Due to the high correlation between PCA_score and patient outcomes, we combined clinical parameters to build a nomogram, which was used to estimate 1-, 3-, and 5-year OS in CRC patients (**Figure 8A**). The calibration curve for this established nomogram showed a high degree of accuracy between the actual observed and predicted values (**Figure 8B**). In addition, we estimated AUC values for these clinical factors to predict OS at 1, 3, and 5 years, respectively. As shown in **Figures 8C, D**, the AUC values implied that this nomogram had excellent predictive value for prognosis as expected.

## Discussion

Cuproptosis is a novel cell death pathway (9). Copper is an indispensable trace element involved in various biological processes. Recent studies have shown that copper levels in serum and tumor tissue are significantly elevated in cancer patients compared to the control group (16, 17). Research evidence suggests ferroptosis may be an adaptive process critical for eradicating cancer-causing cells (18) and that antitumor therapy takes effect by activating ferroptosis (19).

Immunotherapy is an emerging treatment modality for many types of tumors. However, the benefits vary widely from patient to patient. Also, an association between the subgroups of cuproptosis combined with ferroptosis-related genes and immunotherapy, clinical features, and prognosis have not been yet reported. We anticipate that this study will contribute to developing viable CRC immunotherapies.

We identified the transcriptional alterations and expression of cuproptosis and ferroptosis-related genes on the basis of the TCGA–COAD and TCGA–READ cohort in this research. Thirty-three differentially expressed genes were discovered among the 278 cuproptosis and ferroptosis-related genes. The genes ATP7A, DLAT, CDKN2A, ATG5 and ATG7 were core genes through the protein-protein interaction network, which may have an important role in tumorigenesis and treatment. It has been reported that elesclomol alone promotes the degradation of the copper transporter copper transport ATPase 1 (ATP7A), thereby delaying the proliferation of CRC cells (20). The gene CDKN2A was reported to be closely associated with colorectal cancer prognosis (21).

PCA cluster analysis was performed on 33 cuproptosis and ferroptosis-related genes to classify CRC patients into three categories. Most genes are under-expressed in cluster A, and these patients had poor prognoses. However, the genes CDKN2A and GPX4 were highly expressed in cluster A. High expression of GPX4 may inhibit ferroptosis, which in turn promotes the proliferation of cancer cells, resulting in poor prognosis (22), which is consistent with our findings.

The Limma package was used to perform a differential analysis of cuproptosis and ferroptosis-related gene subsets, resulting in 786 common differential genes. It was found that the differential genes were enriched in metabolism, tricarboxylic acid cycle enzyme complex, oxidative phosphorylation and other pathways through GO and KEGG enrichment analysis. Univariate prognostic analysis was further performed on 786 differential genes, and 231 genes associated with prognosis were obtained. PCA_score was established to quantify prognosis-related differential gene subsets by the PCA method. Patients with low PCA_score had unfavorable OS, suggesting a low PCA_score could predict a poor prognosis. We also found that PD-L1 and CTLA-4 were highly expressed in the low PCA_score group, and the difference was statistically significant, which is consistent with the research reported by Seidel et al. (23).

A common immunomodulatory mAb is an immune checkpoint inhibitor (ICI). Cytotoxic T lymphocyte-associated antigen 4 (CTLA-4), one of the immune checkpoints, acts as a co-inhibitory receptor on the surface of T cells (24). CTLA-4 acts as negative regulatory feedback in T cell stimulation, inhibits CD28 binding to MHC and suppresses T cell immune responses (25). Binding of programmed death 1 receptor to ligand PF-L1 mediates potent inhibitory signals to impede T effector cell proliferation and adversely affect antiviral and antitumor immunity (26). Therefore, patients with high expression of PD-L1 and CTLA-4 have a poor prognosis, which is consistent with our findings. Inhibition of PD-L1 and CTLA-4 is beneficial to the treatment of patients with colorectal cancer.

Based on the survival analysis of PCA_score and clinical information, we constructed a nomogram for estimating 1-, 3-, and 5-year OS. No study has been reported to predict the prognosis of colorectal cancer using the cuproptosis and ferroptosis-related genes cluster, and the differential gene PCA_score associated with prognosis. The PCA_score was calculated by the differential genes associated with the prognosis from the cuproptosis and ferroptosis-related genes clusters, and no research on predicting the prognosis of colorectal cancer has been reported. The AUC values can quantify the discriminative power of the prognostic nomogram. The 1-, 3-, and 5-year AUCs for the nomogram of OS ranged from 0.672 to 0.733. The calibration curve of the nomogram fits well with the 45-degree line, illustrating the agreement between the predictive value and actual observations of OS at 1, 3, and 5 years. In conclusion, these nomograms provide a more practical tool to help clinicians develop appropriate individualized treatment regimens for CRC patients, contributing to improving clinical outcomes.

Although the prognostic nomogram is well validated, our study has several limitations. Data from public databases were obtained retrospectively, and inherent selection bias may affect their robustness. In addition, extensive prospective studies and complementary *in vivo* and *in vitro* experimental studies are warranted to validate the efficacy of immunomodulatory mAbs in different subtypes of patients.

## Conclusion

We used the TCGA and GEO databases to determine the association of prognostic differential gene score (PAC_score) with clinical information and immunomodulatory monoclonal antibodies (PD-L1 and CTLA4) and established a new nomograms to estimate 1-, 3-, and 5-year OS. The results showd that the nomogram has satisfactory predictive performance and can be used as a reliable tool for evaluating the prognosis of CRC patients. Furthermore, inhibiting the expression of PD-L1 and CTLA4 has a positive effect on prolonging the survival time of patients.

## Data availability statement

Publicly available datasets were analyzed in this study. This data can be found here: https://portal.gdc.cancer.gov/GSE39582.

## Author contributions

HM designed this research. YL and YD performed data collection and analysis. All of the authors critically reviewed the manuscript and all authors contributed to the article and approved the submitted version.

## Funding

This work was supported by the Tianjin Key Medical Discipline (Specialty) Construction Project and 2020 Tianjin Health Science and Technology Project, Science and Technology Talent Cultivation Project (KJ20009).

## Conflict of interest

The authors declare that the research was conducted without any commercial or financial relationships that could be construed as a potential conflict of interest.

## Publisher’s note

All claims expressed in this article are solely those of the authors and do not necessarily represent those of their affiliated organizations, or those of the publisher, the editors and the reviewers. Any product that may be evaluated in this article, or claim that may be made by its manufacturer, is not guaranteed or endorsed by the publisher.
